# Increased risk of severe COVID-19 in hospitalized patients with SARS-CoV-2 Alpha variant infection: a multicentre matched cohort study

**DOI:** 10.1186/s12879-022-07508-x

**Published:** 2022-06-13

**Authors:** Guillaume Martin-Blondel, François-Xavier Lescure, Lambert Assoumou, Charlotte Charpentier, Jean-Marc Chapplain, Thomas Perpoint, Gaspard Grouteau, Hugues Cordel, Gilles Pialoux, Jérome Pacanowski, Michael Thy, Adeline Bauvois, Didier Laureillard, Fadia Hamrouni, Michèle Algarte-Genin, Julien Poissy, Diane Descamps, Dominique Costagliola, for the CoCliCo Study Group, Guillaume Martin-Blondel, Pierre Delobel, Gaspard Grouteau, Jean Roch Le Henaff, Vincent Mear, Sandra Lagarrigues, Alais Frelat, Thomas De Nadai, Zara Steinmeyer, Arnaud Del Bello, Stéphanie Ruiz, Benjamine Sarton, Elise Noel-Savina, Jacques Izopet, Nathan Peiffer-Smadja, Michael Thy, Mathilde Gare, Diane Le Pluart, François-Xavier Lescure, Christophe Rioux, Laurène Deconinck, Yazdan Yazdanpanah, BenoitVisseaux, Diane Descamps, Charlotte Charpentier, Jean-Marc Chapplain, Pierre Tattevin, Thomas Perpoint, Maude Bouscambert-Duchamp, Hodane Yonis, Paul Chabert, Hugues Cordel, Youssouf Mohamed-Kassim, Nolan Hassold, Segolène Brichler, Julien Caliez, Thomas Rambaud, Marilucy Lopez-Sublet, Frédéric Adnet, Gilles Pialoux, Christia Palacios, Marwa bachir, Marine Nadal, Mathieu turpin, Antoine Parrot, Djeneba Fofana, Jérome Pacanowski, Karine Lacombe, Emmanuelle Gras, Laura Levi, Laure Surgers, Ines Devred, Nadia Valin, Thibault Chiarabini, Jean Luc Meynard, Adeline Bauvois, Clara Duran, Elyanne Gault, Jean-Emmanuel Kahn, Elisabeth Rouveix, Guillaume Geri, Didier laureillard, Albert Sotto, Paul Loubet, Claire Roger, Julien Poissy, Marc Lambert, Ady Assaf, Laurence Bocket, Firouzé Bani-Sadr, Yohan N’Guyen, Juliette Romaru, Maxime Hentzien, Thomas Gabas, Amélie Chabrol, Cecilia Billiou, Philippe Menager, Christophe Billy, Jean-Jacques Laurichesse, Fabrice Ketty N. Simba, Pauline Caraux Paz, Liliane Tinang, Agathe Bounhiol, Catherine Burnat, Sandrine Soriot-Thomas, Damien Basille, Jean Philippe Lanoix, Yoan Zerbib, Yoann Zerbib, Anne Pouvaret, Fanny Lanternier, Helene Mascitti, Aurélien Dinh, Benjamin Davido, Philippe Lesprit, Suresnes, France, Service de Biologie Clinique : Philippe Lesprit, Eric Farfour, Mathilde Neuville, Linda Nait Allaoua, Michèle Lejaille, Nathalie De Castro, Jean-Michel Molina, Diane Ponscarme, Mariagrazia Tateo, Geoffroy Liegeon, Ines Boussen, Pauline Huriez, André Cabié, Valentine Campana, Isabelle Calmont, Jean-Marie Turmel, Guitteaud Karine, Pierre-François Sandrine, Athéna Marquise, Ornella Cabras, Mélanie Lehoux, Cyrille Chabartier, Vincent Dubee, Diama Ndiaye, Caroline Lefeuvre, Achille Kouatchet, Duc Nguyen, Camille Tumiotto, Pierre Sioniac, Alexandre Boyer, Jean-François Faucher, Edouard Desvaux, Sylvie Rogez, Paul Le Turnier, François Raffi, Emmanuel Canet, Antoine Roquilly, Louise Castain, Solène Secher, Véronique Mondain, Lionel Piroth, Christelle Auvray, Pascal Chavanet, Marielle Buisson, Sophie Mahy, François-Xavier Catherine, Clementine Esteve, Michel Duong, Carole Charles, Sandrine Gohier, Céline Schaffer, Olivier Robineau, Perrine Bortolotti, Maxime Pradier, Francois Goehringer, Alice Corbel, Jeanne Kotzyba, Kévin Alexandre, Gaetan Beduneau, Elodie Alessandri-Gradt, Martin Martinot, Simon Gravier, Ciprian Ion, Victoire de Lastours, Roza Rahli, Valérie Garrait, Laurent Richier, Mounira Smati-lafarge, Guillemette Frémont, Pierre Louis Nivose, Marie Hélène André, Magdalena Gerin, Aicha Hamdi, Naomi Sayre, Stephanie Cossec, Sophie Alviset, Pierre Alain Billy, Marie Gousseff, Emmanuel Forestier, Anne-Laure Destrem, Olivier Rogeaux, Alexie Bosch, Sabrina Bryant, Gaëlle Bourgeois, Ophélie Dos Santos Schaller, Marie-Christine Carret, Nicolas Ettahar, Haciba Moudjahed, Nathalie Leone, Mehdi Djennaoui, Nicolas Lefebvre, Axel Ursenbach, François Danion, Yvon Ruch, Morgane Solis, Hamid Merdji, Loïc Kassègne, Fanny Pommeret, Emeline Colomba Blameble, Merad Manssouria, Annabelle Stoclin, Franck Griscelli, Sophie Deriaz, Eric Oziol, Laurent Favier, Julien Obiols, Pascal Gicquel, Christophe Rapp, Laurence Louvet, Paul Ihout, Jean-Benoit Zabbé, Laurent Bellec, Tomasz Chroboczek, Sandrine Mégessier, Marie Lacoste, Benjamin Viala, Thibaut Challan-Belval, Chloé Wackenheim, Paule Letertre-Gibert, Olivier Grossi

**Affiliations:** 1 Service des Maladies Infectieuses et Tropicales, Centre Hospitalier Universitaire de Toulouse, Toulouse, France; 2 Institut Toulousain des Maladies Infectieuses et Inflammatoires (Infinity), INSERM UMR1291, CNRS UMR5051, Université Toulouse III, Toulouse, France; 3 Service des Maladies Infectieuses et Tropicales, Hôpital Bichat-Claude-Bernard, APHP, Paris, France; 4 Sorbonne Université, INSERM, Institut Pierre Louis d’Épidémiologie et de Santé Publique (IPLESP), Paris, France; 5 Service de Virologie, Université de Paris, INSERM, IAME, UMR 1137, AP-HP, Hôpital Bichat-Claude Bernard, 75018 Paris, France; 6 Service des Maladies Infectieuses et Tropicales, Centre Hospitalier Universitaire de Rennes, Rennes, France; 7 Service des Maladies Infectieuses et Tropicales, Hospices Civils de Lyon, Lyon, France; 8 Service des Maladies Infectieuses et Tropicales, Hôpital Avicenne, AP-HP, Bobigny, France; 9 Service des Maladies Infectieuses et Tropicales, Hôpital Tenon, APHP, Paris, France; 10 Service des Maladies Infectieuses et Tropicales, Hôpital Saint-Antoine, APHP, Paris, France; 11 Service de Médecine Interne, Hôpital Ambroise Paré, APHP, Boulogne-Billancourt, France; 12 Service des Maladies Infectieuses et Tropicales, Centre Hospitalier Universitaire de Nîmes, Nîmes, France; 13 University of Lille, Inserm U1285, CHU Lille, Pôle de Médecine Intensive Réanimation, CNRS, UMR 8576, UGSF, Unité de Glycobiologie Structurale et Fonctionnelle, 59000 Lille, France

**Keywords:** COVID-19, SARS-CoV-2, Variant of concern Alpha, Severity

## Abstract

**Background:**

The impact of the variant of concern (VOC) Alpha on the severity of COVID-19 has been debated. We report our analysis in France.

**Methods:**

We conducted an exposed/unexposed cohort study with retrospective data collection, comparing patients infected by VOC Alpha to contemporaneous patients infected by historical lineages. Participants were matched on age (± 2.5 years), sex and region of hospitalization. The primary endpoint was the proportion of hospitalized participants with severe COVID-19, defined as a WHO-scale > 5 or by the need of a non-rebreather mask, occurring up to day 29 after admission. We used a logistic regression model stratified on each matched pair and accounting for factors known to be associated with the severity of the disease.

**Results:**

We included 650 pairs of patients hospitalized between Jan 1, 2021, and Feb 28, 2021, in 47 hospitals. Median age was 70 years and 61.3% of participants were male. The proportion of participants with comorbidities was high in both groups (85.0% vs 90%, p = 0.004). Infection by VOC Alpha was associated with a higher odds of severe COVID-19 (41.7% vs 38.5%—aOR = 1.33 95% CI [1.03–1.72]).

**Conclusion:**

Infection by the VOC Alpha was associated with a higher odds of severe COVID-19.

**Supplementary Information:**

The online version contains supplementary material available at 10.1186/s12879-022-07508-x.

## Background

Since the end of 2020, the SARS-CoV-2 variant of concern (VOC) Alpha, also known as B.1.1.7 or VOC-202012/01 has rapidly spread across all continents [1, 2]. It has been shown that the VOC Alpha is between 43 and 90% more transmissible than variants from historical lineages (HL) 19A/B and 20A/B/C/D/E/F/G [3–6]. The effect of the VOC Alpha on COVID-19 severity is less clear, although some authors, mostly from the United Kingdom, reported an increased risk of hospitalization [7, 8] or of mortality [9–12], while others did not report changes in either symptoms, disease duration [13], or severity [14]. Variation in study designs and settings, particularly when medical infrastructures are strained, may explain these discrepancies.

In France, the VOC Alpha accounted for 3.3% of the viruses sequenced on January 8th, 2021, and reached 83% on April 15th, 2021 [15]. Therefore, while the two first epidemic waves affecting France in 2020 were related to HL, the current epidemic occurring since January 2021 is characterized by a progressive overlapping switch towards VOC Alpha dominance. The aim of this study was to assess the effect of VOC Alpha compared to HL on COVID-19 severity in a multicentre matched exposed and unexposed cohort study with retrospective data collection focusing on patients admitted to the hospital during a time when both the HL and the VOC Alpha coexisted and while there was no limitation in medical resources.

## Methods

All adults (age > 18 years) hospitalized for symptomatic acute COVID-19 between Jan 1, 2021, and Feb 28, 2021, with a positive VOC Alpha screening were eligible for the study. Over the same time period, the maximum number of patients concurrently hospitalized for COVID-19 in France was 24,820, including 3492 patients in ICU, which is lower than the maximal bed capacity (108,183 beds in medical wards, including 5433 beds in ICU, and 5954 additional beds in intensive care) [16]. During the time period of the study, COVID-19 diagnosis through PCR on nasopharyngeal sampling was widely and freely available to everyone. VOC Alpha screening was performed using the ThermoFischer kit (TaqPath One-step RT-qPCR, ThermoFischer Scientific, Waltham, MA, USA) with Spike gene target RT-PCR failure or mutations-specific real-time RT-PCR (i.e. deletion 69–70 and N501Y mutation, TIB Molbiol, Berlin, Germany). The combination of spike deletion at residue 69–70 and N501Y mutation was interpreted as a suspicion of VOC Alpha. All eligible participants who objected to the use of their data were excluded from the analyses. Participants with VOC Alpha were matched in a 1:1 ratio to HL on the basis of age (± 2.5 years), sex and administrative region of hospitalization.

### Data collection

Data were retrospectively collected from all participating sites of the CoCliCo (Collective of COVID-19 clinicians) network. All sites were asked to identify all adults who met the eligibility criteria and collect the site number, age and sex of these patients for centralized matching. The list of matched participants was then sent to the sites to fill out the electronic case report form. Data relevant to the study’s objectives were extracted from the patients’ medical records. Data on COVID-19 vaccination status were not collected in the study at a time where only 2.4% of the eligible population (above 75 years of age or healthcare workers) had received a complete vaccine scheme*.*

### Outcomes

The primary outcome was the proportion of participants with a severe form of COVID-19 occurring up to day 29 after the date of hospitalization. Severity was defined by a WHO clinical progression scale > 5 (high flow oxygen therapy (HFOT), non-invasive ventilation (NIV), invasive ventilation, extra-corporeal membrane oxygenation (ECMO) or death) [17] but also by the need of a non-rebreather mask (NRB) in order to consider patients with severe COVID-19 but limitations of life-sustaining treatment. Two participants with missing primary outcome were considered as having a severe form.

The secondary endpoints were: (i) mortality; (ii) WHO clinical progression scale > 5; (iii) admission to an intensive care unit (ICU); (iv) invasive ventilation or ECMO; (v) HFOT, all up to day 29, (vi) time from symptom onset to hospitalization, and (vii) re-admission after discharge up to day 29. Maximal parenchymal lesions extension and pulmonary thrombo-embolism detected on chest CT-scan were collected.

### Statistical analyses

For an 80% power, a 5% type I error and an expected severity of 20% in patients infected with HL, 1100 exposed and 1100 unexposed individuals to VOC Alpha were needed to detect a 25% higher risk of severity in participants infected with VOC Alpha compared to HL with 1:1 matching, while the number of matched pairs corresponding to 30%, 40% and 50% higher risk of severity were 769, 444, and 291 respectively.

The analysis population consisted of all individuals exposed to VOC Alpha with a matched unexposed control. The characteristics of exposed and unexposed individuals to VOC Alpha were compared with a Mc Nemar test for categorical variables and Wilcoxon paired test for continuous variables.

Unadjusted and adjusted odds ratios (OR) were calculated using a logistic regression model stratified on each matched pair to assess the association between VOC Alpha infection and the occurrence of a severe form of COVID-19. The following factors associated with COVID-19 severity were accounted for in multivariable models: age, BMI, smoking and comorbidities (cardiovascular disease, chronic lung disease, asthma, chronic kidney disease, chronic liver disease, chronic neurological disease, active cancer, solid organ or hematopoietic cell transplantation, autoimmune disease, HIV infection, and diabetes). Treatments received during the course of hospitalization could be the result of a worse course of the disease and therefore be on the causal pathway of a more severe disease; therefore, receiving corticosteroids was not included in the main model.

Time-to-event methods, including Kaplan–Meier estimates and Cox proportional-hazards models, were used to analyze all secondary outcomes. Unadjusted and adjusted hazard ratios (HR) were calculated using Cox proportional hazard model stratified on each pair to assess whether VOC Alpha was associated with the outcome. Death was not accounted as a competing risk in the main analysis, and was accounted for in sensitivity analyses for high flow oxygen therapy, ICU admission, and mechanical ventilation or ECMO. Analyses were conducted with SAS software version 9.4 (SAS Statistical Institute, Cary, North Carolina). All statistical tests were 2-tailed, with α = 0.05.

## Results

**Fig. 1:**
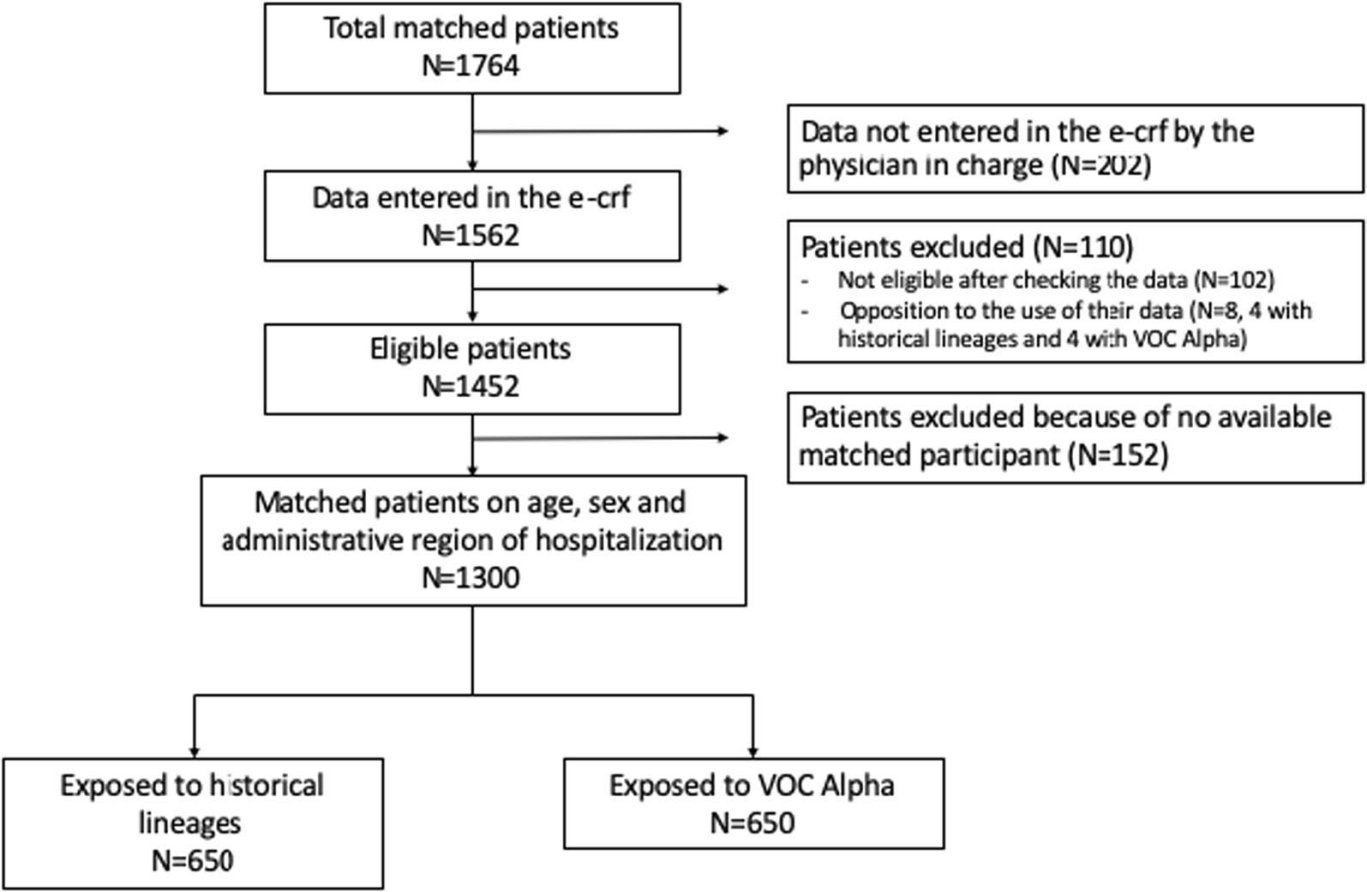
Flow chart

In this multicentre matched exposed-unexposed cohort study, 882 participants with VOC Alpha infection were eligible and 650 were enrolled and matched on the basis of sex, age and administrative region, to 650 contemporaneous participants infected by HL (Fig. 1).

Characteristics of patients are presented in Table 1 according to SARS-CoV-2 lineage. The median age was 70 years (range 25 to 101) and 61.3% were males. Participants with VOC Alpha infection less often had at least one of the specific comorbidities listed above, and were less often smokers, than their matched participants. The proportion of patients first admitted in the ICU did not differ between groups (16.7% in the VOC Alpha group vs 13.8% in the HL group, P = 0.12). Median oxygen saturation level before initiation of oxygen therapy was not different (90% (range 32–99) in the VOC Alpha group vs 91% (range 30–99) in the HL group, P = 0.12), as well as the acme of C-reactive protein level during the first three days of hospitalization (median 93 mg/L (range 1–584) in the VOC Alpha group vs 89 mg/L (range 1–423) in the HL group, P = 0.34). Proportions of patients who received corticosteroids and other immunomodulatory therapies were higher in the VOC Alpha group (respectively 84.3% and 8.4%) than in the HL group (respectively 76.6%, P < 0.001 and 5.2%, P < 0.02). The proportion of participants who received anticoagulant and antibiotic therapy did not differ (93.3% versus 91.3% and 60.3 versus 60.6% respectively). Only 5 participants in each group received monoclonal antibodies or antiviral drugs.

**Table 1:** Participants characteristics at hospital admission

	Exposed to historical lineages	Exposed to VOC Alpha	P-value
	N = 650	N = 650	
Age (years), median (range)	70 (27–100)	70 (25–101)	
Gender—n (%)			
Male	399 (61.4)	399 (61.4)	
Female	251 (38.6)	251 (38.6)	
Body Mass Index			0.20
N	571	553	
Median (range)	26.8 (13.5–67.2)	27.4 (3.3–76)	
Smoker—n (%)	169 (26.0)	136 (20.9)	0.04
Location of initial care—n (%)			0.12
Conventional hospitalization	560 (86.2)	539 (82.9)	
ICU	90 (13.8)	109 (16.7)	
Comorbidities, n (%)	586 (90.1)	553 (85.0)	0.004
Cardiovascular disease	410 (62.9)	401 (61.6)	0.57
Chronic lung disease	102 (15.6)	74 (11.3)	0.02
Asthma	35 (5.3)	33 (5.0)	1.00
Chronic kidney disease	87 (13.3)	70 (10.7)	0.11
Chronic liver disease	21 (3.2)	12 (1.8)	0.11
Chronic neurological disease	115 (17.6)	88 (13.5)	0.03
Active cancer	80 (12.3)	53 (8.1)	0.01
Solid organ transplant	31 (4.7)	13 (2.0)	< 0.001
Autoimmune disease	27 (4.1)	20 (3.0)	0.15
HIV Infection	10 (1.5)	0 (0)	
Obesity	164 (25.2)	176 (27.0)	0.54
Diabetes	197 (30.3)	182 (27.9)	0.29

The proportion of severe COVID-19 (defined as a WHO score > 5 or the need of a NRB) within 29 days of hospitalization was 41.7% in the VOC Alpha group and 38.5% in the HL group (aOR 1.33 95% confidence interval (95% CI): [1.02–1.72] in the multivariable analysis) and similar results were observed for the 2 components of the primary endpoints (Table 2). Regarding secondary outcomes within 29 days after hospitalization, the mortality rate was 24% in the VOC Alpha group and 19% in the HL group (aHR 1.21 [0.93–1.58] in the multivariable analysis), and the proportion of patients reaching a WHO score > 5 was 26.2% in the VOC Alpha group and 20.5% in the HL group (aHR 1.24 [1.00–1.55] in the multivariable analysis). All other secondary endpoints were not significantly associated with infection by the VOC Alpha. The entire multivariable model and Kaplan–Meier curves are provided as Additional file 1.

**Table 2:** Primary and secondary outcomes

	Exposed to historical lineages N = 650	Exposed to VOC Alpha N = 650	Crude measure of association (95% CI)	Adjusted measure of association (95% CI)
*WHO scale > 5 or non-rebreather mask at day 29*				
Number of events by Day 29	250	271		
Proportion of participants with an event at Day 29—% (95% CI)	38.5 (34.7–42.3)	41.7 (37.8–45.6)	1.15 (0.91–1.45)	1.33 (1.03–1.72)
*Mortality rate at day 29*				
Number of deaths by Day 29	112	130		
Kaplan–Meier estimate of mortality by Day 29—% (95% CI)	19.0 (16.3–23.8)	24.0 (20.9–29.4)	1.18 (0.92–1.52)	1.21 (0.93–1.58)
*WHO scale > 5 by day 29*				
Number of participants with WHO scale > 5 by Day 29	164	179		
Kaplan–Meier estimate of WHO scale > 5 by Day 29—% (95% CI)	20.5 (17.0–24.7)	26.2 (22.2–30.8)	1.19 (0.96–1.48)	1.24 (1.00–1.55)
*Non-rebreather mask by day 29*				
Number of participants with non-rebreather mask by Day 29	177	208		
Kaplan–Meier estimate of non-rebreather mask by Day 29—% (95% CI)	29.7 (26.0–33.8)	35.2 (31.3–39.5)	1.18 (0.97–1.45)	1.20 (0.98–1.47)
High flow oxygen therapy by day 29*				
Number of high flow oxygen therapy by Day 29	201	240		
Kaplan–Meier estimate of oxygen therapy by Day 29—% (95% CI)	35.4 (31.3–39.9)	42.1 (37.8–46.6)	1.20 (0.99–1.44)	1.18 (0.98–1.40)
ICU admission by day 29*				
Number of ICU admission by Day 29	207	240		
Kaplan–Meier estimate of ICU admission by Day 29—% (95% CI)	36.0 (32.0–40.0)	41.7 (37.6–46.1)	1.15 (0.96–1.39)	1.12 (0.93–1.36)
Mechanical ventilation or ECMO by day 29*				
Number of Mechanical ventilation or ECMO by Day 29	108	107		
Kaplan–Meier estimate of Mechanical ventilation or ECMO by Day 29—% (95% CI)	20.6 (17.0–24.7)	21.5 (17.4–25.7)	0.97 (0.74–1.27)	0.96 (0.73–1.27)
*Hospitalization*				
Median time from symptoms onset to hospitalization (95% CI)—days	6 (5–6)	7 (6–7)	0.93 (0.83–1.04)	0.96 (0.86–1.08)
Median duration on hospitalization (95% CI)—days	11.5 (10–13)	11.0 (10–12)	1.02 (0.88–1.18)	0.95 (0.81–1.10)
*Readmission*				
Number of readmissions after previous discharge	28	19		
Kaplan–Meier estimate of re-admission after a discharge—% (95% CI)	5.1 (3.4–7.7)	2.3 (1.2–4.3)	0.68 (0.38–1.23)	(0.42–1.42)

^*^In an analysis accounting for competing risk of death, the adjusted sHR were estimated as 1.17 (0.97–1.41), 1.09 (0.89–1.35) and 0.96 (0.73–1.25) respectively

Acme of parenchymal extent on chest CT-scan was higher in the VOC Alpha group (50%, range 0–95) than in the HL group (40%, range 0–99, univariable analysis P = 0.04). The proportion of patients diagnosed with pulmonary embolism did not differ (5.3% in the VOC Alpha group, and 6.0% in the HL group, univariable analysis P = 0.29).

## Discussion

In this multicentre matched exposed-unexposed cohort study, we found a 33% (95%CI: 3–72%) higher odds of severe COVID-19 in participants infected by a VOC Alpha, while the increase in the risk of death within 29 days after hospitalization was not significant (21% (95% CI: − 7% to + 58%).

These results are in line with the literature showing an increased severity of VOC Alpha compared to HL. The European Surveillance System analyzed 19,207 cases of VOC Alpha and 3,348 HL cases reported between Sept 14, 2020, and March 14, 2021, from seven European countries [7]. In this study, patients infected with VOC Alpha were found to have a 1.7 times higher risk of being hospitalized for COVID-19. Hospitalized patients were also shown to be younger (by 10 years in median) and to be less comorbid than patients hospitalized for COVID-19 related to HL in this study and others [7, 11, 14]. The latter was also true in our study. Comparison of data concerning the age of infected patients is more complex because of a different epidemiological context and vaccine strategies, hence we do not provide additional data on that matter, given that participants were matched on age in our study. We can however underline that the impact of VOC Alpha on the higher occurrence of severe COVID-19 is reinforced by the fact that our patients have less comorbidities than the matched non-exposed patients.

The effect of VOC Alpha on the risk of mortality is still the subject of debate. Both the study from the OpenSAFELY electronic health records [11] and three community-based studies [9, 10, 12] performed in the United Kingdom from Oct 1, 2020, to Feb 14, 2021, which compared VOC Alpha to HL, showed an increased hazard of death of 1.55 to 1.67. These studies could have been biased by the epidemiological context and overwhelmed hospital capacities, that may have increased the impact of COVID-19 in the most severely ill patients. Of note, although our study may have been underpowered to detect a significant increase in the risk of death, the confidence interval of the mortality hazard in our study is compatible with the reported confidence intervals reported in the United Kingdom with a higher bound of 1.58. Conversely, rates of ICU admission or death did not differ significantly in any age group in the study from The European Surveillance System [7]. In addition, there was no evidence of an association between severe disease, death and lineage in a hospital-based cohort study of patients acutely admitted to hospitals in London from Nov 9, 2020, to Dec 20, 2020, before the peak of hospital admissions [14], but that study was small and the baseline date was different for participants with symptoms (date of symptoms) and those without (date of hospitalization). These discrepancies between studies underline the need to consider the geographical area, and the potential impact of the epidemiological pressure on healthcare facilities that could have increased morbimortality of COVID-19.

Why VOC Alpha is associated with an increased severity in human beings is unknown. Our study highlights clinically relevant details depicting the course and pathogenesis of COVID-19 related to VOC Alpha. Patients with VOC Alpha infection were not hospitalized sooner after the onset of first symptoms, and were not more frequently admitted to ICU first than patients infected with HL. However, we found a higher maximal parenchymal extent of ground-glass opacities on chest CT-scan. In the meantime, these patients were more likely to reach a WHO score > 5. This suggests that increased pathogenicity of VOC Alpha is not linked with a shortened delay between the first symptoms and the hospital admission. Patients were hospitalized at the beginning of their second week of symptoms, at a time which is considered to be the “inflammatory phase” of the disease. At the same time, high nasopharyngeal viral loads are central to pathogenesis of viral infections and have been shown in SARS to be associated with the onset of symptoms, oxygen desaturation, mechanical ventilation, and death [18]. Recent studies showed that COVID-19 patients infected by VOC Alpha had a viral load 3 to 10 times higher than the HL in nasopharyngeal samples [14, 19, 20]. This higher viral load in SARS-CoV-2 VOC Alpha infection can result from a higher virus binding affinity to the angiotensin-converting enzyme 2 receptor [21], which likely enhances entry to epithelial host cells in the respiratory tract and the lungs and could trigger a stronger immune response causing a more severe disease compared to HL. This might be illustrated by the higher parenchymal extent of ground-glass opacities on chest CT-scan, although the acme of C-reactive protein serum level during the first three days of hospitalization did not differ between groups. In the present study, SARS-CoV-2 nasopharyngeal viral loads, estimated by real-time PCR Ct values, were not recorded due to the heterogeneity of RT-PCR assays used in this multicentre study, which renders difficult their interpretations due to the inter-assays variations.

In this study we controlled for several potential confounding factors by using a short study period and matching on the basis of administrative region to account for the potential impact of the local burden of the epidemic on the care system which can influence the clinical outcomes. Given the strong effect of age on the severity of the disease we matched participants exposed to the VOC Alpha to participant exposed to HL on age within 2.5 years and we also adjusted the analysis according to age. We also accounted for the presence of comorbidities and smoking, factors known to be associated with a more severe course of the disease. Only 650 of the 882 patients infected with VOC Alpha initially listed as eligible by the clinical sites could finally be enrolled and matched. Although it is unlikely for this drop to be strongly linked with the outcomes, we cannot exclude some selection bias. Socio-economic status and origin were not collected and could not be accounted for, although they are associated with severity of disease. Given the retrospective nature of data collection, we had to restrict data collection to variables available in medical records of most participants. For instance, although obesity was accounted for, we could not collect the exact BMI which would have been more precise. As in any observational studies, the remaining role of additional confounders cannot be ruled out. Finally, we considered the results of SARS-CoV-2 screening test strategies and not of viral genome sequencing, however a very high level of agreement has been described in the literature between the presence of deletion 69–70 and the VOC Alpha [9].

## Conclusion

VOC Alpha is associated with an increased severity, and potentially leads to an increased mortality. These considerations have huge implications for vaccine allocation policies. Vaccination should now urgently be made accessible to patients who were not previously prioritized in order to reach herd immunity. These results also point to the importance of limiting the circulation of the virus until a very large proportion of the population is vaccinated.

## Supplementary Information

**Additional file 1: Table S1.** Unadjusted and adjusted analysis of factors associated with COVID-19 severity by Day 29 using a stratified logistic regression model on each matched pair. **Table S2.** Multivariable analysis of factors associated with mortality by Day 29 using a stratified Cox regression model on each matched pair. **Table S3.** Multivariable analysis of factors associated with WHO scale >5 by Day 29 using a stratified Cox regression model on each matched pair. **Table S4. **Multivariable analysis of factors associated with non-rebreather mask by Day 29 using a stratified Cox regression model on each matched pair. **Table S5. **Multivariable analysis of factors associated with high flow oxygen therapy by day 29 using a stratified Cox regression model on each matched pair. **Table S6. **Multivariable analysis of factors associated with ICU admission by day 29 using a stratified Cox regression model on each matched pair. **Table S7. **Multivariable analysis of factors associated with Mechanical ventilation or ECMO by day 29 using a stratified Cox regression model on each matched pair. **Table S8. **Multivariable analysis of factors associated with time from symptoms onset to hospitalization using a stratified Cox regression model on each matched pair. **Table S9. **Multivariable analysis of factors associated with duration on hospitalization using a stratified Cox regression model on each matched pair. **Table S10. **Multivariable analysis of factors associated with readmission using a stratified Cox regression model on each matched pair. **Figure S1.** Kaplan–Meir plot for all cause of mortality. **Figure S2.** Kaplan–Meir plot for WHO scale >5. **Figure S3.** Kaplan–Meir plot for non-rebreather mask. **Figure S4.** Kaplan–Meir plot for high flow oxygen therapy. **Figure S5. **Kaplan–Meir plot for intensive care admission. **Figure S6. **Kaplan–Meir plot for Mechanical ventilation or ECMO. **Figure S7. **Kaplan–Meir plot for hospitalization (time from symptoms onset to hospitalization). **Figure S8. **Kaplan–Meir plot for hospital discharge (duration on hospitalization). **Figure S9. **Kaplan–Meir plot for readmission.

## Data Availability

Data requests should be sent to Prof Dominique Costagliola. Data access must be approved by the French data protection authority, la Commission Nationale de l’Informatique et des Libertés. For further information, please see: https://www.cnil.fr/.

## Abbreviations

VOC: Variant of concern
COVID-19: Coronavirus disease-19
SARS-CoV-2: Severe acute respiratory syndrome coronavirus 2
WHO: World Health Organization
aOR: Adjusted odds ratios
HR: Hazard ratios
HL: Historical lineages
CoCliCo: Collective of COVID-19 clinicians
HFOT: High flow oxygen therapy
NIV: Non-invasive ventilation
ECMO: Extra-corporeal membrane oxygenation
NRB: Non-rebreather mask
