# Supplementary material for: Increased risk of severe COVID-19 in hospitalized patients with SARS-CoV-2 Alpha variant infection: a multicentre matched cohort study
Source: BMC Infect Dis. 2022 Jun 13;22:540. doi: 10.1186/s12879-022-07508-x (PMC9189443; doi:10.1186/s12879-022-07508-x)
Supplement: Supplementary file 1 — Additional file 1: Table S1. Unadjusted and adjusted analysis of factors associated with COVID-19 severity by Day 29 using a stratified logistic regression model on each matched pair. Table S2. Multivariable analysis of factors associated with mortality by Day 29 using a stratified Cox regression model on each matched pair. Table S3. Multivariable analysis of factors associated with WHO scale >5 by Day 29 using a stratified Cox regression model on each matched pair. Table S4. Multivariable analysis of factors associated with non-rebreather mask by Day 29 using a stratified Cox regression model on each matched pair. Table S5. Multivariable analysis of factors associated with high flow oxygen therapy by day 29 using a stratified Cox regression model on each matched pair. Table S6. Multivariable analysis of factors associated with ICU admission by day 29 using a stratified Cox regression model on each matched pair. Table S7. Multivariable analysis of factors associated with Mechanical ventilation or ECMO by day 29 using a stratified Cox regression model on each matched pair. Table S8. Multivariable analysis of factors associated with time from symptoms onset to hospitalization using a stratified Cox regression model on each matched pair. Table S9. Multivariable analysis of factors associated with duration on hospitalization using a stratified Cox regression model on each matched pair. Table S10. Multivariable analysis of factors associated with readmission using a stratified Cox regression model on each matched pair. Figure S1. Kaplan–Meir plot for all cause of mortality. Figure S2. Kaplan–Meir plot for WHO scale >5. Figure S3. Kaplan–Meir plot for non-rebreather mask. Figure S4. Kaplan–Meir plot for high flow oxygen therapy. Figure S5. Kaplan–Meir plot for intensive care admission. Figure S6. Kaplan–Meir plot for Mechanical ventilation or ECMO. Figure S7. Kaplan–Meir plot for hospitalization (time from symptoms onset to hospitalization). Figure S8. Kaplan–Meir plot fo [file 12879_2022_7508_MOESM1_ESM.docx]

**Additional Results**

**Table S1**: Unadjusted and adjusted analysis of factors associated with COVID-19 severity by Day 29 using a stratified logistic regression model on each matched pair

|  |  |  |  | **Univariate analysis** | **Multivariate analysis** |
| --- | --- | --- | --- | --- | --- |
|  |  | N of patients | N of event (%) | Odds Ratio (95% CI) | Odds Ratio (95% CI) |
| **Covid Variant** |  |  |  |  |  |
|  | Historical lineages | 650 | 250 (38.5) | 1 | 1 |
|  | VOC Alpha | 650 | 271 (41.7) | 1.15 (0.91 - 1.45) | 1.33 (1.03 - 1.72) |
| **Age, years** |  |  |  |  |  |
|  | <60 | 333 | 89 (26.7) | 1 | 1 |
|  | (60 - 64) | 157 | 63 (40.1) | 1.07 (0.37 - 3.07) | 0.94 (0.27 - 3.02) |
|  | (65 - 69) | 138 | 61 (44.2) | 1.16 (0.27 - 4.99) | 1.33 (0.28 - 6.33) |
|  | (70 - 74) | 184 | 96 (52.2) | 1.66 (0.33 - 8.17) | 1.77 (0.32 - 9.58) |
|  | 75+ | 488 | 212 (43.4) | 1.59 (0.25 - 10.17) | 1.78 (0.25 - 12.59) |
| **Body Mass Index (BMI, kg/m^2^)** |  |  |  |  |  |
|  | <18.5 | 42 | 14 (33.3) | 1 | 1 |
|  | (18.5 - 25) | 516 | 179 (34.7) | 1.44 (0 .52 -4.01) | 1.79 (0 .78 -5.61) |
|  | (25.01 -29.99) | 369 | 143 (38.8) | 1.45 (0.49 -4.23) | 1.93 (0.57 -6.44) |
|  | 30+ | 373 | 185 (49.6) | 2.65 (0.94 -7.47) | 3.57 (1.10 -11.61) |
| **Heart disease** |  |  |  |  |  |
|  | No | 489 | 166 (33.9) | 1 | 1 |
|  | Yes | 811 | 355 (43.8) | 1.23 (0.84 -1.79) | 0.91 (0.59 -1.41) |
| **Chronic lung disease** |  |  |  |  |  |
|  | No | 1124 | 433 (38.5) | 1 | 1 |
|  | Yes | 176 | 88 (50) | 1.14 (0.72 -1.81) | 0.79 (0.46 -1.38) |
| **Asthma** |  |  |  |  |  |
|  | No | 1232 | 496 (40.3) | 1 | 1 |
|  | Yes | 68 | 25 (36.8) | 1.03 (0.51 -2.06) | 1.07 (0.50 -2.30) |
| **Chronic kidney disease** |  |  |  |  |  |
|  | No | 1143 | 442 (38.7) | 1 | 1 |
|  | Yes | 157 | 79 (50.3) | 1.25 (0.78 -2.0) | 0.99 (0.56 -1.74) |
| **Chronic liver disease** |  |  |  |  |  |
|  | No | 1267 | 506 (39.9) | 1 | 1 |
|  | Yes | 33 | 15 (45.5) | 1.96 (0.72 -5.36) | 0.67 (0.22 -2.08) |
| **Chronic neurological disease** |  |  |  |  |  |
|  | No | 1097 | 430 (39.2) | 1 | 1 |
|  | Yes | 203 | 91 (44.8) | 1.57 (0.99 -2.49) | 0.47 (0.28 -0.80) |
| **Active cancer** |  |  |  |  |  |
|  | No | 1167 | 451 (38.6) | 1 | 1 |
|  | Yes | 133 | 70 (52.6) | 1.82 (1.04 -3.16) | 0.44 (0.24 -0.84) |
| **Solid organ or hematopoietic cell transplant** |  |  |  |  |  |
|  | No | 1256 | 505 (40.2) | 1 | 1 |
|  | Yes | 44 | 16 (36.4) | 1.11 (0.45 -2.73) | 0.57 (0.20 -1.64) |
| **Autoimmune disease** |  |  |  |  |  |
|  | No | 1253 | 499 (39.8) | 1 | 1 |
|  | Yes | 47 | 22 (46.8) | 1.00 (0.40 -2.48) | 1.08 (0.41 -2.85) |
| **HIV Infection** |  |  |  |  |  |
|  | No | 1290 | 519 (40.2) | 1 | 1 |
|  | Yes | 10 | 2 (20) | 2.0 (0.18 -22) | 0.41 (0.03 -4.76) |
| **Diabetes** |  |  |  |  |  |
|  | No | 921 | 338 (36.7) | 1 | 1 |
|  | Yes | 379 | 183 (48.3) | 1.63 (1.11 -2.39) | 0.70 (0.46 -1.09) |
| **Tabacco** |  |  |  |  |  |
|  | No | 994 | 384 (38.6) | 1 | 1 |
|  | Yes | 305 | 137 (44.9) | 1.20 (0.82 -1.77) | 0.83 (0.53 -1.30) |
| **Dexamethasone or other corticosroids** |  |  |  |  |  |
|  | No | 252 | 40 (15.9) | 1 | 1 |
|  | Yes | 1048 | 481 (45.9) | 5.22 (2.98 -9.14) | 6.30 (3.37 -11.80) |
|  |  |  |  |  |  |

**Table S2:** Multivariable analysis of factors associated with mortality by Day 29 using a stratified Cox regression model on each matched pair

|  |  |  |  | **Multivariate analysis** |
| --- | --- | --- | --- | --- |
|  |  | N of patients | N of Event | Hazard ratio (95% CI) |
| **Covid Variant** |  |  |  |  |
|  | Historical lineages | 650 | 112 | 1 |
|  | VOC Alpha | 650 | 130 | 1.21 (0.93 -1.58) |
| **Sex** |  |  |  |  |
|  | Female | 502 | 91 | 1 |
|  | Male | 798 | 151 | 1.21 (0.92 -1.61) |
| **Age, years** |  |  |  |  |
|  | <60 | 333 | 20 | 1 |
|  | (60 - 64) | 157 | 15 | 1.17 (0.59 -2.32) |
|  | (65 - 69) | 138 | 17 | 1.45 (0.75 -2.82) |
|  | (70 - 74) | 184 | 28 | 1.59 (0.87 -2.89) |
|  | 75+ | 488 | 162 | 4.85 (2.91 -8.06) |
| **BMI, kg/m^2^** |  |  |  |  |
|  | <18.5 | 42 | 11 | 1 |
|  | (18.5 - 25) | 516 | 107 | 0.94 (0.50 - 1.76) |
|  | (25.01 -29.99) | 369 | 50 | 0.68 (0.35 -1.32) |
|  | 30+ | 373 | 74 | 1.29 (0.67 -2.48) |
| **Heart disease** |  |  |  |  |
|  | No | 489 | 50 | 1 |
|  | Yes | 811 | 192 | 1.35 (0.96 -1.89) |
| **Chronic lung disease** |  |  |  |  |
|  | No | 1124 | 188 | 1 |
|  | Yes | 176 | 54 | 1.72 (1.23 -2.40) |
| **Asthma** |  |  |  |  |
|  | No | 1232 | 232 | 1 |
|  | Yes | 68 | 10 | 0.55 (0.28 -1.06) |
| **Chronic kidney disease** |  |  |  |  |
|  | No | 1143 | 187 | 1 |
|  | Yes | 157 | 55 | 1.60 (1.14 -2.24) |
| **Chronic liver disease** |  |  |  |  |
|  | No | 1267 | 235 | 1 |
|  | Yes | 33 | 7 | 1.04 (0.48 -2.25) |
| **Chronic neurological disease** |  |  |  |  |
|  | No | 1097 | 176 | 1 |
|  | Yes | 203 | 66 | 1.59 (1.16 -2.16) |
| **Active cancer** |  |  |  |  |
|  | No | 1167 | 199 | 1 |
|  | Yes | 133 | 43 | 2.1 (1.50 -3.07) |
|  |  |  |  |  |
| **Solid organ or hematopoietic cell transplant** |  |  |  |  |
|  | No | 1256 | 236 | 1 |
|  | Yes | 44 | 6 | 0.70 (0.30 -1.66) |
| **Autoimmune disease** |  |  |  |  |
|  | No | 1253 | 232 | 1 |
|  | Yes | 47 | 10 | 1.22 (0.63 -2.36) |
| **HIV Infection** |  |  |  |  |
|  | No | 1290 | 240 | 1 |
|  | Yes | 10 | 2 | 1.75(0.42 -7.21) |
| **Diabetes** |  |  |  |  |
|  | No | 921 | 152 | 1 |
|  | Yes | 379 | 90 | 1.13 (0.85 -1.50) |
| **Tabacco** |  |  |  |  |
|  | No | 994 | 182 | 1 |
|  | Yes | 305 | 60 | 0.94 (0.68 -1.31) |

**Table S3:** Multivariable analysis of factors associated with WHO scale >5 by Day 29 using a stratified Cox regression model on each matched pair

|  |  |  |  | **Multivariate analysis** |
| --- | --- | --- | --- | --- |
|  |  | N of patients | N of Event | Hazard ratio (95% CI) |
| **Covid Variant** |  |  |  |  |
|  | Historical lineages | 650 | 164 | 1 |
|  | VOC Alpha | 650 | 179 | 1.24 (1.00 -1.55) |
| **Sex** |  |  |  |  |
|  | Female | 502 | 197 | 1 |
|  | Male | 798 | 379 | 1.37 (1.08 -1.74) |
| **Age, years** |  |  |  |  |
|  | <60 | 333 | 47 | 1 |
|  | (60 - 64) | 157 | 37 | 1.38 (0.88 - 2.15) |
|  | (65 - 69) | 138 | 33 | 1.23 (0.78 -1.95) |
|  | (70 - 74) | 184 | 54 | 1.49 (0.98 -2.25) |
|  | 75+ | 488 | 171 | 2.95(2.05 -4.25) |
| **BMI, kg/m2** |  |  |  |  |
|  | <18.5 | 42 | 11 | 1 |
|  | (18.5 - 25) | 516 | 122 | 1.11 (0.59 - 2.07) |
|  | (25.01 -29.99) | 369 | 84 | 1.03 (0.54 -1.95) |
|  | 30+ | 373 | 246 | 1.69 (0.89 -3.18) |
| **Heart disease** |  |  |  |  |
|  | No | 489 | 91 | 1 |
|  | Yes | 811 | 251 | 1.16 (0.89 -1.51) |
| **Chronic lung disease** |  |  |  |  |
|  | No | 1124 | 254 | 1 |
|  | Yes | 176 | 88 | 1.55 (1.15 -2.08) |
| **Asthma** |  |  |  |  |
|  | No | 1232 | 325 | 1 |
|  | Yes | 68 | 17 | 0.67 (0.40 -1.11) |
| **Chronic kidney disease** |  |  |  |  |
|  | No | 1143 | 277 | 1 |
|  | Yes | 157 | 65 | 1.27 (0.94 -1.73) |
| **Chronic liver disease** |  |  |  |  |
|  | No | 1267 | 333 | 1 |
|  | Yes | 33 | 9 | 0.81 (0.41 -1.60) |
| **Chronic neurological disease** |  |  |  |  |
|  | No | 1097 | 273 | 1 |
|  | Yes | 203 | 69 | 1.45 (1.08 -1.94) |
| **Active cancer** |  |  |  |  |
|  | No | 1167 | 292 | 1 |
|  | Yes | 133 | 50 | 1.75 (1.27 -2.42) |
| **Solid organ or hematopoietic cell transplant** |  |  |  |  |
|  | No | 1256 | 328 | 1 |
|  | Yes | 44 | 14 | 1.17 (0.65 -2.10) |
| **Autoimmune disease** |  |  |  |  |
|  | No | 1253 | 328 | 1 |
|  | Yes | 47 | 14 | 1.08 (0.61 -1.89) |
| **HIV Infection** |  |  |  |  |
|  | No | 1290 | 340 | 1 |
|  | Yes | 10 | 2 | 1.22 (0.30 -50) |
| **Diabetes** |  |  |  |  |
|  | No | 921 | 212 | 1 |
|  | Yes | 379 | 130 | 1.18 (0.93 -1.50) |
| **Tabacco** |  |  |  |  |
|  | No | 994 | 251 | 1 |
|  | Yes | 305 | 91 | 1.05 (0.81 -1.38) |
|  |  |  |  |  |

**Table S4:** Multivariable analysis of factors associated with non-rebreather mask by Day 29 using a stratified Cox regression model on each matched pair

|  |  |  |  | **Multivariate analysis** |
| --- | --- | --- | --- | --- |
|  |  | N of patients | N of event | Hazard ratio (95% CI) |
| **Covid Variant** |  |  |  |  |
|  | Historical lineages | 650 | 177 | 1 |
|  | VOC Alpha | 650 | 208 | 1.20 (0.98 -1.47) |
| **Sex** |  |  |  |  |
|  | Female | 502 | 149 | 1 |
|  | Male | 798 | 236 | 0.95 (0.76 - 1.17) |
| **Age, years** |  |  |  |  |
|  | <60 | 333 | 67 | 1 |
|  | (60 - 64) | 157 | 44 | 1.35 (0.91 -1.99) |
|  | (65 - 69) | 138 | 44 | 1.53 (1.04 -2.26) |
|  | (70 - 74) | 184 | 73 | 2.07 (1.42 -2.87) |
|  | 75+ | 488 | 157 | 1.87 (1.35 -2.60) |
| **BMI, kg/m^2^** |  |  |  |  |
|  | <18.5 | 42 | 10 |  |
|  | (18.5 - 25) | 516 | 127 | 1.18 (0.61 -2.27) |
|  | (25.01 -29.99) | 369 | 109 | 1.57(0.81 -3.05) |
|  | 30+ | 373 | 139 | 2.19(1.13 -4.24) |
| **Heart disease** |  |  |  |  |
|  | No | 489 | 126 | 1 |
|  | Yes | 811 | 259 | 1.02(0.80 -1.30) |
| **Chronic lung disease** |  |  |  |  |
|  | No | 1124 | 327 | 1 |
|  | Yes | 176 | 58 | 1.06 (0.79 -1.43) |
| **Asthma** |  |  |  |  |
|  | No | 1232 | 496 | 1 |
|  | Yes | 68 | 25 | 0.92 (0.59 -1.45) |
| **Chronic kidney disease** |  |  |  |  |
|  | No | 1143 | 330 | 1 |
|  | Yes | 157 | 55 | 1.18 (0.86 -1.60) |
| **Chronic liver disease** |  |  |  |  |
|  | No | 1267 | 376 | 1 |
|  | Yes | 33 | 9 | 0.80 (0.41 -1.57) |
| **Chronic neurological disease** |  |  |  |  |
|  | No | 1097 | 321 | 1 |
|  | Yes | 203 | 64 | 1.15 (0.86 -1.53) |
| **Active cancer** |  |  |  |  |
|  | No | 1167 | 333 | 1 |
|  | Yes | 133 | 52 | 1.47 (1.08 -2.00) |
|  |  |  |  |  |
| **Solid organ or hematopoietic cell transplant** |  |  |  |  |
|  | No | 1256 | 378 | 1 |
|  | Yes | 44 | 7 | 0.54 (0.25 -1.17) |
| **Autoimmune disease** |  |  |  |  |
|  | No | 1253 | 369 | 1 |
|  | Yes | 47 | 16 | 1.16 (0.69 -1.95) |
| **HIV Infection** |  |  |  |  |
|  | No | 1290 | 384 | 1 |
|  | Yes | 10 | 1 | 0.42 (0.05 -3.02) |
| **Diabetes** |  |  |  |  |
|  | No | 921 | 263 | 1 |
|  | Yes | 379 | 122 | 1.04(0.83 -1.31) |
| **Tabacco** |  |  |  |  |
|  | No | 994 | 286 | 1 |
|  | Yes | 305 | 99 | 1.08 (0.84 -1.38) |
|  |  |  |  |  |

**Table S5:** Multivariable analysis of factors associated with high flow oxygen therapy by day 29 using a stratified Cox regression model on each matched pair

|  |  |  |  | **Multivariate analysis** |
| --- | --- | --- | --- | --- |
|  |  | N of patients | N of Event | Hazard ratio (95% CI) |
| **Covid Variant** |  |  |  |  |
|  | Historical lineages | 650 | 201 | 1 |
|  | VOC Alpha | 650 | 240 | 1.18 (0.98 – 1.40) |
| **Sex** |  |  |  |  |
|  | Female | 502 | 393 | 1 |
|  | Male | 798 | 658 | 1.12 (0.98 - 1.28) |
| **Age, years** |  |  |  |  |
|  | <60 | 333 | 269 | 1 |
|  | (60 - 64) | 157 | 128 | 0.87 (0.70 - 1.09) |
|  | (65 - 69) | 138 | 112 | 0.78 (0.62 -0.98) |
|  | (70 - 74) | 184 | 145 | 0.79 (0.64 -0.98) |
|  | 75+ | 488 | 397 | 1.10 (0.92 -1.32) |
| **BMI, kg/m2** |  |  |  |  |
|  | <18.5 | 42 | 31 | 1 |
|  | (18.5 - 25) | 516 | 414 | 1.14 (0.78 - 1.65) |
|  | (25.01 -29.99) | 369 | 303 | 1.08 (0.74 -1.58) |
|  | 30+ | 373 | 303 | 1.01 (0.69 -1.48) |
| **Heart disease** |  |  |  |  |
|  | No | 489 | 385 | 1 |
|  | Yes | 811 | 666 | 1.07 (0.93 -1.27) |
| **Chronic lung disease** |  |  |  |  |
|  | No | 1124 | 908 | 1 |
|  | Yes | 176 | 143 | 1.16(0.96 -1.40) |
| **Asthma** |  |  |  |  |
|  | No | 1232 | 997 | 1 |
|  | Yes | 68 | 54 | 0.94(0.71 -1.24) |
| **Chronic kidney disease** |  |  |  |  |
|  | No | 1143 | 933 | 1 |
|  | Yes | 157 | 118 | 0.95(0.77 -1.18) |
| **Chronic liver disease** |  |  |  |  |
|  | No | 1267 | 1024 | 1 |
|  | Yes | 33 | 27 | 0.97(0.66 -1.44) |
| **Chronic neurological disease** |  |  |  |  |
|  | No | 1097 | 887 | 1 |
|  | Yes | 203 | 164 | 1.09 (0.91 -1.31) |
| **Active cancer** |  |  |  |  |
|  | No | 1167 | 956 | 1 |
|  | Yes | 133 | 95 | 0.86 (0.69 -1.07) |
| **Solid organ or hematopoietic cell transplant** |  |  |  |  |
|  | No | 1256 | 1021 | 1 |
|  | Yes | 44 | 30 | 0.64 (0.43 -0.95) |
| **Autoimmune disease** |  |  |  |  |
|  | No | 1253 | 1014 | 1 |
|  | Yes | 47 | 37 | 0.85 (0.61 -1.19) |
| **HIV Infection** |  |  |  |  |
|  | No | 1290 | 1043 | 1 |
|  | Yes | 10 | 8 | 0.84 (0.41 -1.70) |
| **Diabetes** |  |  |  |  |
|  | No | 921 | 743 | 1 |
|  | Yes | 379 | 308 | 0.94 (0.82 -1.09) |
| **Tabacco** |  |  |  |  |
|  | No | 994 | 800 | 1 |
|  | Yes | 305 | 251 | 0.99 (0.85 -1.16) |

**Table S6:** Multivariable analysis of factors associated with ICU admission by day 29 using a stratified Cox regression model on each matched pair

|  |  |  |  | **Multivariate analysis** |
| --- | --- | --- | --- | --- |
|  |  | N of patients | N of Event | Hazard ratio (95% CI) |
| **Covid Variant** |  |  |  |  |
|  | Historical lineages | 650 | 207 | 1 |
|  | VOC Alpha | 650 | 240 | 1.12 (0.93 -1.36) |
| **Sex** |  |  |  |  |
|  | Female | 502 | 140 | 1 |
|  | Male | 798 | 300 | 1.48 (1.20 -1.83) |
| **Age, years** |  |  |  |  |
|  | <60 | 333 | 119 | 1 |
|  | (60 - 64) | 157 | 70 | 1.25 (0.91 - 1.68 ) |
|  | (65 - 69) | 138 | 55 | 1.02 (0.76 -1.49) |
|  | (70 - 74) | 184 | 87 | 1.49 (1.10 -2.01) |
|  | 75+ | 488 | 111 | 0.82 (0.61 -1.10) |
| **BMI; kg/m^2^** |  |  |  |  |
|  | <18.5 | 42 | 5 | 1 |
|  | (18.5 - 25) | 516 | 121 | 1.57(1.07 - 2.31) |
|  | (25.01 -29.99) | 369 | 150 | 2.33 (1.62 -3.34) |
|  | 30+ | 373 | 164 | 2.59 (1.80 -3.72) |
| **Heart disease** |  |  |  |  |
|  | No | 489 | 165 | 1 |
|  | Yes | 811 | 275 | 1.07 (0.86 -1.34) |
| **Chronic lung disease** |  |  |  |  |
|  | No | 1124 | 374 | 1 |
|  | Yes | 176 | 66 | 1.38 (1.04 -1.83) |
| **Asthma** |  |  |  |  |
|  | No | 1232 | 416 | 1 |
|  | Yes | 68 | 24 | 1.00 (0.66 -1.52) |
| **Chronic kidney disease** |  |  |  |  |
|  | No | 1143 | 397 | 1 |
|  | Yes | 157 | 43 | 0.75 (0.53 -1.06) |
| **Chronic liver disease** |  |  |  |  |
|  | No | 1267 | 425 | 1 |
|  | Yes | 33 | 15 | 1.21 (0.71 -2.05) |
| **Chronic neurological disease** |  |  |  |  |
|  | No | 1097 | 406 | 1 |
|  | Yes | 203 | 34 | 0.50 (0.35 -0.73) |
| **Active cancer** |  |  |  |  |
|  | No | 1167 | 394 | 1 |
|  | Yes | 133 | 46 | 0.92 (0.66 -1.27) |
|  |  |  |  |  |
| **Solid organ or hematopoietic cell transplant** |  |  |  |  |
|  | No | 1256 | 428 | 1 |
|  | Yes | 44 | 12 | 0.78 (0.42 -1.44) |
| **Autoimmune disease** |  |  |  |  |
|  | No | 1253 | 414 | 1 |
|  | Yes | 47 | 22 | 1.66 (1.07 -2.57) |
| **HIV Infection** |  |  |  |  |
|  | No | 1290 | 435 | 1 |
|  | Yes | 10 | 5 | 2(0.91 -5.52) |
| **Diabetes** |  |  |  |  |
|  | No | 921 | 301 | 1 |
|  | Yes | 379 | 139 | 1.17(0.94 -1.45) |
| **Tabacco** |  |  |  |  |
|  | No | 994 | 334 | 1 |
|  | Yes | 305 | 106 | 0.80 (0.63 -1.01) |

**Table S7:** Multivariable analysis of factors associated with Mechanical ventilation or ECMO by day 29 using a stratified Cox regression model on each matched pair

|  |  |  |  | **Multivariate analysis** |
| --- | --- | --- | --- | --- |
|  |  | N of patients | N of Event | Hazard ratio (95% CI) |
| **Covid Variant** |  |  |  |  |
|  | Historical lineages | 650 | 108 | 1 |
|  | VOC Alpha | 650 | 107 | 0.96 (0.73 -1.27) |
| **Sex** |  |  |  |  |
|  | Female | 502 | 145 | 1 |
|  | Male | 798 | 300 | 1.34 (1.09 -1.66) |
| **Age, years** |  |  |  |  |
|  | <60 | 333 | 133 | 1 |
|  | (60 - 64) | 157 | 77 | 1.23 (0.92 -1.65) |
|  | (65 - 69) | 138 | 58 | 0.98 (0.71 -1 .35) |
|  | (70 - 74) | 184 | 86 | 1.36 (1.01 -1.82) |
|  | 75+ | 488 | 91 | 0.60 (0.44 -0.81) |
| **BMI, kg/m2** |  |  |  |  |
|  | <18.5 | 42 | 4 | 1 |
|  | (18.5 - 25) | 516 | 122 | 2.77 (1.01 - 7.61) |
|  | (25.01 -29.99) | 369 | 139 | 3.93 (1.43 -10.80) |
|  | 30+ | 373 | 180 | 5.27 (1.92 -14.46) |
| **Heart disease** |  |  |  |  |
|  | No | 489 | 171 | 1 |
|  | Yes | 811 | 274 | 1.09 (0.88 -1.36) |
| **Chronic lung disease** |  |  |  |  |
|  | No | 1124 | 392 | 1 |
|  | Yes | 176 | 53 | 0.88 (0.65 -1.20) |
| **Asthma** |  |  |  |  |
|  | No | 1232 | 422 | 1 |
|  | Yes | 68 | 23 | 0.90 (0.58 -1.38) |
| **Chronic kidney disease** |  |  |  |  |
|  | No | 1143 | 401 | 1 |
|  | Yes | 157 | 44 | 0.90 (0.64 -1.26) |
| **Chronic liver disease** |  |  |  |  |
|  | No | 1267 | 430 | 1 |
|  | Yes | 33 | 15 | 1.21 (0.71 -2.05) |
| **Chronic neurological disease** |  |  |  |  |
|  | No | 1097 | 415 | 1 |
|  | Yes | 203 | 30 | 0.47 (0.32 -0.70) |
| **Active cancer** |  |  |  |  |
|  | No | 1167 | 405 | 1 |
|  | Yes | 133 | 40 | 0.85 (0.61 -1.20) |
| **Solid organ or hematopoietic cell transplant** |  |  |  |  |
|  | No | 1256 | 433 | 1 |
|  | Yes | 44 | 12 | 0.69 (0.37 -1.30) |
| **Autoimmune disease** |  |  |  |  |
|  | No | 1253 | 418 | 1 |
|  | Yes | 47 | 27 | 2.00 (1.33 -3.00) |
| **HIV Infection** |  |  |  |  |
|  | No | 1290 | 441 | 1 |
|  | Yes | 10 | 5 | 1.46 (0.53 -4.00) |
| **Diabetes** |  |  |  |  |
|  | No | 921 | 305 | 1 |
|  | Yes | 379 | 140 | 1.16 (0.93 -1.40) |
| **Tabacco** |  |  |  |  |
|  | No | 994 | 322 | 1 |
|  | Yes | 305 | 123 | 1.17 (0.89 -1.39) |
|  |  |  |  |  |

**Table S8:** Multivariable analysis of factors associated with time from symptoms onset to hospitalization using a stratified Cox regression model on each matched pair

|  |  |  |  | **Multivariate analysis** |
| --- | --- | --- | --- | --- |
|  |  | N of patients | N of events | Hazard Ratio (95% CI) |
| **Covid Variant** |  |  |  |  |
|  | Historical lineages | 650 | 650 | 1 |
| Hazard Ratio (95% CI) | VOC Alpha | 650 | 650 | 0.96 (0.96 -1.08) |
| **Sex** |  |  |  |  |
|  | Female | 502 | 502 | 1 |
| Hazard Ratio (95% CI) | Male | 798 | 798 | 1.01 (0.89-1.13) |
| **Age, years** |  |  |  |  |
|  | <60 | 333 | 333 | 1 |
|  | (60 - 64) | 157 | 157 | 1.13 (0.93- 1.38) |
|  | (65 - 69) | 138 | 138 | 0.93 (0.75-1.14) |
|  | (70 - 74) | 184 | 184 | 1.09 (0.89-1.32) |
|  | 75+ | 488 | 488 | 1.44 (1.22-1.70) |
| **BMI, kg/m2** |  |  |  |  |
|  | <18.5 | 42 | 42 | 1 |
|  | (18.5 - 25) | 516 | 516 | 0.67 (0.49- 0.93) |
|  | (25.01 -29.99) | 369 | 369 | 0.67 (0.48-0.93) |
|  | 30+ | 373 | 373 | 0.76 (0.55-1.07) |
| **Heart disease** |  |  |  |  |
|  | No | 489 | 489 | 1 |
|  | Yes | 811 | 811 | 0.98 (0.86-1.12) |
| **Chronic lung disease** |  |  |  |  |
|  | No | 1124 | 1124 | 1 |
|  | Yes | 176 | 176 | 1.23 (1.03-1.45) |
| **Asthma** |  |  |  |  |
|  | No | 1232 | 1232 | 1 |
|  | Yes | 68 | 62 | 0.87 (0.68-1.12) |
| **Chronic kidney disease** |  |  |  |  |
|  | No | 1143 | 1143 | 1 |
|  | Yes | 157 | 157 | 1.35 (1.12-1.63) |
| **Chronic liver disease** |  |  |  |  |
|  | No | 1267 | 1267 | 1 |
|  | Yes | 33 | 33 | 0.78 (0.55-1.12) |
| **Chronic neurological disease** |  |  |  |  |
|  | No | 1097 | 1097 | 1 |
|  | Yes | 203 | 203 | 1.44 (1.22-1.70) |
| **Active cancer** |  |  |  |  |
|  | No | 1167 | 1167 | 1 |
|  | Yes | 133 | 133 | 1.18 (0.82-1.43) |
| **Solid organ or hematopoietic cell transplant** |  |  |  |  |
|  | No | 1256 | 1256 | 1 |
|  | Yes | 44 | 44 | 1.15 (0.82-1.61) |
| **Autoimmune disease** |  |  |  |  |
|  | No | 1253 | 1253 | 1 |
|  | Yes | 47 | 47 | 0.85 (0.62-1.15) |
| **HIV Infection** |  |  |  |  |
|  | No | 1290 | 1290 | 1 |
|  | Yes | 10 | 10 | 0.96 (0.48-1.88) |
| **Diabetes** |  |  |  |  |
|  | No | 921 | 921 | 1 |
|  | Yes | 379 | 379 | 1.00 (0.87-1.14) |
| **Tabacco** |  |  |  |  |
|  | No | 994 | 994 | 1 |
|  | Yes | 305 | 305 | 1.04 (0.91-1.20) |

**Table S9:** Multivariable analysis of factors associated with duration on hospitalization using a stratified Cox regression model on each matched pair

|  |  |  |  | **Multivariate analysis** |
| --- | --- | --- | --- | --- |
| Variable |  | N of patients | N of events | Hazard ratio (95% CI) |
| **Covid Variant** |  |  |  |  |
|  | Historical lineages | 649 | 349 | 1 |
|  | VOC Alpha | 649 | 354 | 0.95 (0.81-1.10) |
| **Sex** |  |  |  |  |
|  | Female | 501 | 373 | 1 |
|  | Male | 797 | 425 | 0.88 (0.75-1.03) |
| **Age, years** |  |  |  |  |
|  | <60 | 332 | 119 | 1 |
|  | (60 - 64) | 157 | 70 | 0.64 (0.50- 0.82) |
|  | (65 - 69) | 138 | 55 | 0.59 (0.46-0.77) |
|  | (70 - 74) | 184 | 87 | 0.45 (0.35-0.59) |
|  | 75+ | 487 | 111 | 0.42 (0.34-0.53) |
| **BMI, kg/m3** |  |  |  |  |
|  | <18.5 | 41 | 16 | 1 |
|  | (18.5 - 25) | 516 | 281 | 1.50 (0.90- 2.50) |
|  | (25.01 -29.99) | 369 | 224 | 1.52 (0.90-2.55) |
|  | 30+ | 372 | 182 | 0.97 (0.57-1.64) |
| **Heart disease** |  |  |  |  |
|  | No | 488 | 310 | 1 |
|  | Yes | 810 | 393 | 1.06 (0.89-1.27) |
| **Chronic lung disease** |  |  |  |  |
|  | No | 1123 | 628 | 1 |
|  | Yes | 175 | 75 | 0.95 (0.74-1.23) |
| **Asthma** |  |  |  |  |
|  | No | 1230 | 664 | 1 |
|  | Yes | 68 | 39 | 1.01 (0.72-1.41) |
| **Chronic kidney disease** |  |  |  |  |
|  | No | 1143 | 645 | 1 |
|  | Yes | 155 | 58 | 0.71 (0.52-0.96) |
| **Chronic liver disease** |  |  |  |  |
|  | No | 1265 | 685 | 1 |
|  | Yes | 33 | 18 | 0.93 (0.57-1.50) |
| **Chronic neurological disease** |  |  |  |  |
|  | No | 1097 | 623 | 1 |
|  | Yes | 203 | 80 | 0.76 (0.59-0.97) |
| **Active cancer** |  |  |  |  |
|  | No | 1165 | 653 | 1 |
|  | Yes | 133 | 50 | 0.65 (0.48-0.88) |
| **Solid organ or hematopoietic cell transplant** |  |  |  |  |
|  | No | 1254 | 677 | 1 |
|  | Yes | 44 | 26 | 0.85 (0.55-1.31) |
| **Autoimmune disease** |  |  |  |  |
|  | No | 1253 | 687 | 1 |
|  | Yes | 45 | 16 | 0.43 (0.26-0.71) |
| **HIV Infection** |  |  |  |  |
|  | No | 1288 | 698 | 1 |
|  | Yes | 10 | 5 | 0.63 (0.26-1.55) |
| **Diabetes** |  |  |  |  |
|  | No | 920 | 527 | 1 |
|  | Yes | 379 | 176 | 0.81 (0.67-0.97) |
| **Tabacco** |  |  |  |  |
|  | No | 994 | 553 | 1 |
|  | Yes | 304 | 150 | 0.80 (0.66-0.97) |

**Table S10:** Multivariable analysis of factors associated with readmission using a stratified Cox regression model on each matched pair

|  |  |  |  | **Multivariate analysis** |
| --- | --- | --- | --- | --- |
|  |  | N of patients | N of events | Hazard Ratio (95% CI) |
| **Covid Variant** |  |  |  |  |
|  | Historical lineages | 428 | 28 | 1 |
|  | VOC Alpha | 428 | 19 | 0.78 (0.42 -1.42) |
| **Sex** |  |  |  |  |
|  | Female | 336 | 22 | 1 |
| Hazard Ratio (95% CI) | Male | 520 | 25 | 0.54 (0.27-1.06) |
| **Age, years** |  |  |  |  |
|  | <60 | 268 | 14 | 1 |
|  | (60 - 64) | 103 | 6 | 1.07 (0.38- 2.99) |
|  | (65 - 69) | 89 | 4 | 0.91 (0.27-2.98) |
|  | (70 - 74) | 108 | 6 | 1.29 (0.44-3.76) |
|  | 75+ | 288 | 17 | 1.06 (0.41-2.74) |
| **BMI, kg/m2** |  |  |  |  |
|  | <18.5 | 150 | 6 | 1 |
|  | (18.5 - 25) | 243 | 23 | 1.87 (0.67- 5.16) |
|  | (25.01 -29.99) | 261 | 10 | 0.98 (0.31-3.02) |
|  | 30+ | 202 | 8 | 1.24 (0.40-3.83) |
| **Heart disease** |  |  |  |  |
|  | No | 363 | 16 | 1 |
|  | Yes | 490 | 31 | 1.01 (0.46-2.20) |
| **Chronic lung disease** |  |  |  |  |
|  | No | 755 | 45 | 1 |
|  | Yes | 98 | 2 | 2.38 (1.04-5.43) |
| **Asthma** |  |  |  |  |
|  | No | 805 | 38 | 1 |
|  | Yes | 48 | 10 | 1.04 (0.24-4.48) |
| **Chronic kidney disease** |  |  |  |  |
|  | No | 778 | 35 | 1 |
|  | Yes | 75 | 12 | 2.38 (1.04-5.43) |
| **Chronic liver disease** |  |  |  |  |
|  | No | 833 | 46 | 1 |
|  | Yes | 20 | 1 | 4.13 (1.71-9.97) |
| **Chronic neurological disease** |  |  |  |  |
|  | No | 738 | 37 | 1 |
|  | Yes | 115 | 10 | 1.92 (0.83-4.40) |
| **Active cancer** |  |  |  |  |
|  | No | 787 | 44 | 1 |
|  | Yes | 66 | 3 | 0.93 (0.27-3.17) |
| **Solid organ or hematopoietic cell transplant** |  |  |  |  |
|  | No | 827 | 40 | 1 |
|  | Yes | 26 | 7 | 3.25 (1.28-8.24) |
| **Autoimmune disease** |  |  |  |  |
|  | No | 829 | 45 | 1 |
|  | Yes | 24 | 2 | 2.11 (0.48-9.18) |
| **HIV Infection** |  |  |  |  |
|  | No | 848 | 47 | 1 |
|  | Yes | 5 | 0 | NA |
| **Diabetes** |  |  |  |  |
|  | No | 636 | 35 | 1 |
|  | Yes | 217 | 11 | 0.59 (0.27-1.30) |
| **Tabacco** |  |  |  |  |
|  | No | 672 | 38 | 1 |
|  | Yes | 181 | 9 | 0.87 (0.35-2.11) |

Figure S1: Kaplan-Meir plot for all cause of mortality

Figure S2: Kaplan-Meir plot for WHO scale >5

Figure S3: Kaplan-Meir plot for non-rebreather mask

Figure S4: Kaplan-Meir plot for high flow oxygen therapy

**Figure S5:** Kaplan-Meir plot for intensive care admission

**Figure S6:** Kaplan-Meir plot for Mechanical ventilation or ECMO

**Figure S7:** Kaplan-Meir plot for hospitalization (time from symptoms onset to hospitalization)

**Figure S8:** Kaplan-Meir plot for hospital discharge (duration on hospitalization)

**Figure S9:** Kaplan-Meir plot for readmission
